# The Attitudes of Egyptian Web-Based Health Information Seekers Toward Health Information Provided Through the Internet: Qualitative Study

**DOI:** 10.2196/30108

**Published:** 2022-02-18

**Authors:** Mayada Ghweeba, Antje Lindenmeyer, Sobhi Shishi, Amani Waheed, Mostafa Kofi, Shaymaa Amer

**Affiliations:** 1 Institute of Clinical Sciences Birmingham United Kingdom; 2 Community Medicine Department, Faculty of Medicine, Suez Canal University Ismailia Egypt

**Keywords:** Egyptian internet users, online health information, doctor–patient relationship

## Abstract

**Background:**

The internet has become an established source of health information for many Egyptian internet users. Understanding users’ attitudes toward the benefits and limitations of web-based health information will explain the influence of this information on users’ health-related behavior and decisions.

**Objective:**

This qualitative study aims to understand the attitude of Egyptian internet users toward internet health information and to explore the impact of obtained health information on users’ behavior and on their physician-patient relationship.

**Methods:**

For this qualitative study, semistructured interviews were conducted with a total of 49 participants (41/49, 84% Egyptian internet users and 8/49, 16% physicians) who participated in focus groups or individual interviews. We used a thematic analysis approach to explain and demonstrate participants’ views, thoughts, and experiences in using web-based health information.

**Results:**

The internet has become an important source of health information in comparison with other health information sources and is the central theme that has emerged across the thematic analysis. The attitude toward the use of internet health was classified into three main themes: feeling toward web-based health information (with subthemes: favoring, disliking, neutral, or having ambivalence feelings), motivators to seek internet health information, and behavioral changes using internet health information (subthemes: confidence, satisfaction, and improved knowledge). Themes that emerged from physicians’ interviews included the accessibility of the internet health information, good communication, and coordination of care between patients and their physicians, and the active engagement of patients with their management plan.

**Conclusions:**

The internet has become an essential source of health information for Egyptian adults. Internet health information can improve the patient-physician relationship, especially when users discuss the obtained health information with their physician. Internet health information provided seekers with social support and self-confidence when making health decisions.

## Introduction

Searching for online health information (OHI) has become one of the most popular web-based activities among Egyptians [1,2]. However, the use of OHI in developed countries is greater than that in low or middle socioeconomic populations [3]. Traditional modes of health information such as asking local experts or seeking mass media still play pivotal roles in disseminating health information for many health information seekers in developing countries [4]. In Egypt, previous studies have found that women with higher educational achievement, with the presence of children in the household and having chronic health conditions were more likely to search for health information on the internet [5].

The internet provides a wide range of health topics from healthy eating and fitness to information about self-management of chronic health problems [6,7]. The internet presents medical information through a variety of mediums including websites, listservs, online support groups, chat rooms, instant messaging, and emails [8]. In addition, the internet serves as a medium for users to interact frequently with health care professionals, access health information on the web, and participate in online support groups [3,7].

The accessibility and convenience of the OHI motivate users to search for health information through the internet [9,10]. Moreover, OHI has enabled users to find support from people with similar health problems, which had a substantial impact on their physical and psychological well-being. The availability of OHI is known to empower patients toward healthier behavior changes, become active collaborators in their health decisions, and become more adherent to their treatment [11]. Nevertheless, many concerns have been raised about using the OHI, such as the quality, completeness, and accuracy of OHI, especially in the Arabic language, which is difficult to ascertain by OHI users [2,12]. Previous studies have found a direct relationship between OHI seeking and health literacy [13]. They found that OHI seekers should have a minimal level of health literacy and guidance to be able to review and evaluate the quality of the available health information on the internet, particularly in the absence of proper communication with their physician [14,15].

In Egypt, the need for affordable, accessible, and quality health information source is growing in accordance with the growth of the Egyptian population. Although the internet has become an important source of health information for many internet users, little information is available about Egyptians’ OHI-seeking behavior. In this study, we used a qualitative method to understand how and why people seek OHI and explore the influence of this information on users’ behavior and their relationship with their physicians. The qualitative approach allowed for in-depth insights into the participants’ experiences, behavior, and feelings associated with health-seeking behaviors to better recognize their needs and identify the required improvements in the area of internet health information quality and availability [16].

## Methods

### Ethical Approval, Study Design, and Participants

Participants of this qualitative study were drawn from Egyptian internet users who participated in our previous cross-sectional study to identify the personal characteristics of Egyptian OHI seekers [1]. The inclusion criteria for the study were being Egyptian, aged ≥18 years, living in Egypt at the time of the study, and previously used the internet to search for health information for themselves or others. A total of 49 participants (41/49, 84% Egyptian internet users and 8/49, 16% physicians) participated in 9 focus group discussions (FGDs) and 11 individual interviews. The 8 physicians who participated in this study were purposively selected from physicians who had at least 1 year of cumulative experience of providing OHI through different health information websites. They all received a summary of the study objectives and an explanation of the qualitative study procedures. The FGDs were held at different community locations convenient to the participants; participants’ travel expenses were reimbursed. The 11 interviews, including physicians’ interviews, were conducted through telecommunications apps such as Skype or phone calls.

Consent was obtained from all participants before the FGD and the interviews commenced; participants were reassured that their data would be anonymized. They had the option to choose a nickname to be used during the interviews and data analysis. At the beginning of the FGD and the interviews, participants were asked to complete a brief questionnaire that included demographic information (age, gender, marital status, level of education, current occupation, and self-assessed general health status) and questions about health information-seeking methods such as their preferred source of health information and the frequency of seeking OHI.

Semistructured interviews with open-ended questions were conducted to allow the participants to explain and demonstrate their views, thoughts, and experiences in their own words. We used 2 interview topic guides, one for internet users and another for physicians, which were revised iteratively, allowing for further exploration of new issues raised [10]. The interview guides started with a warm-up question about participants’ perception of the common health topics people are looking for on the internet. The interview guide included questions about the frequency of OHI seeking, influence of web-based interaction with others who have similar health concerns, personal reasons that encourage or prevent them from seeking OHI, perceived impact of OHI-seeking activities on the relationship with their physicians, and how they personally assessed the quality and reliability of health information on the internet. Similarly, the physicians’ interview started with a demographic questionnaire about their gender, years in practice, their medical specialty, and any health information website they used, followed by open-ended questions to obtain insights from their experience of the benefits and challenges of OHI, OHI seekers’ motives and challenges, and how OHI might affect the physician-patient relationship. Probes were used according to the interview topic guides when the participants’ narratives ended or deviated significantly from the topic of interest.

The duration of the FGDs ranged between 56 and 82 (mean 68.7, SD 7.5) minutes, and the range for the interviews was 22 and 46 (mean 34.3, SD 7.1) minutes.

The main data sources were the transcribed audio-recorded interviews, the notes summary collected in each interview, and the questionnaire form used at the beginning of the interviews.

Ethical approval for the study was obtained from the Medical Research Ethics Committee of the Faculty of Medicine at Suez Canal University and granted approval for conducting this study on January 8, 2017 (approval #2298).

### Analysis

Questionnaire results were analyzed to provide descriptive statistics for the demographic characteristics and general use of OHI. Data were analyzed using SPSS Statistics (version 25, IBM Corp) predictive analytics software. Qualitative data were managed and analyzed manually using a thematic analysis approach [16]. The first stage involved translation into English, which included cultural and interpretive insights, and then transcription of all the recorded interviews. Transcripts and notes were read repeatedly to focus on preserving as much detail as possible. The transcripts were then systematically coded. The initial codes were sorted into themes and subthemes, and then these themes were re-evaluated (merged, deleted, or refined) to ensure that each theme had sufficient supporting data and the data cohered meaningfully. The data were then displayed in a graphic format to organize the data and show connections between different themes [17].

## Results

### Overview

As previously outlined, a total of 49 participants (41/49, 84% Egyptian OHI seekers and 8/49, 16% physicians) took part in the study. The demographic characteristics of the OHI seekers are shown in Table 1. The age of the OHI seekers ranged between 19 and 57 years, with a mean age of 36.3 years. Table 1 shows that 63% (26/41) of the OHI seekers were women and 73% (30/41) participants had a university degree. Most of the OHI seekers had health insurance coverage (28/41, 68%), and 17% (7/41) participants had chronic health conditions. Nearly half of the OHI seekers (21/41, 51%) rated their general health status as good.

Regarding the physicians who participated in this study (n=8), 5 (63%) participants were men and 4 (50%) participants had more than 10 years of medical practice, whereas most of the participants had 1 to 2 years of experience in providing OHI through health websites (Table 2).

**Figure 1:**
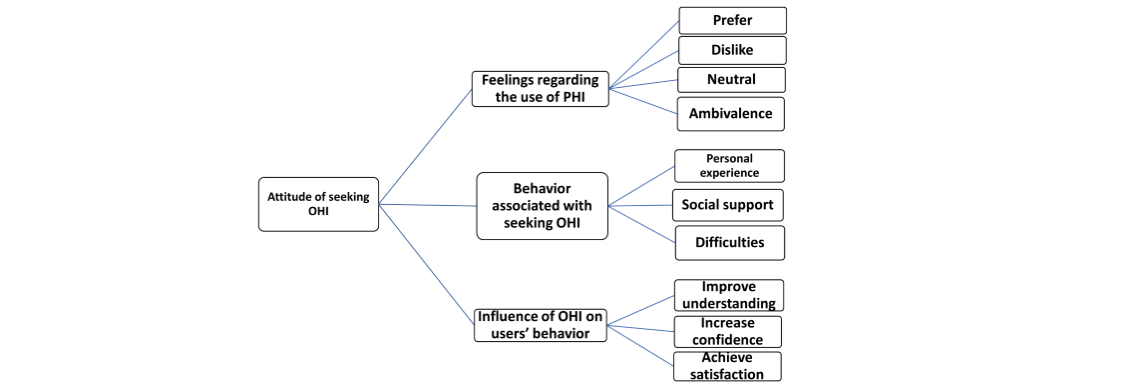
The attitude of Egyptian online health information seekers toward using internet health information. OHI: online health information; PHI: personal health information.

Analysis of the qualitative data showed that *the use of the internet as a source of health information has grown in comparison to other health information sources* has emerged as a central or main theme across all the interviews. We used a graphic framework to summarize our findings into three major themes: participants’ feelings, OHI-seeking behavior, and the influence of OHI on users’ health and their relationship with their physicians (Figure 1). Although each theme was presented and expanded separately, they were often linked to each other owing to the complexity of the research topic. The extracts were cited to illustrate the range of participants’ responses for each theme.

**Table 1:** Demographic and internet use information of the online health information seekers (n=41 internet users).

Sociodemographic characteristics			Value, n (%)
**Age (years)**			
	15-34	17 (41)	
	35-49	18 (44)	
	≥50	6 (15)	
**Gender**			
	Male	15 (37)	
	Female	26 (63)	
**Level of education**			
	University student	7 (17)	
	University graduate	30 (73)	
	Postgraduate degree	4 (10)	
**Social status**			
	Never married before	9 (22)	
	Married	28 (68)	
	Divorced, widow, or widower	4 (10)	
**Employment**			
	Employed	20 (49)	
	Unemployed^a^	14 (34)	
	Student	7 (17)	
**Self-reported general health status**			
	Excellent or very good	21 (51)	
	Good or fair	18 (44)	
	Poor	2 (5)	
**Having a chronic health condition**			
	Yes^b^	7 (17)	
	No	34 (83)	
**Having health insurance coverage**			
	Yes	28 (68)	
	No	13 (32)	

^a^Unemployed also included housewife or retired.

^b^Having one or more chronic health problem.

**Table 2:** Demographic characteristics of the physicians (n=8 physicians).

Characteristics of the physicians			Value, n (%)
**Gender**			
	Male	5 (63)	
	Female	3 (37)	
**Years in practice**			
	0-5	2 (25)	
	6-10	2 (25)	
	>10	4 (50)	
**Medical specialty**			
	Internal medicine	4 (50)	
	Family medicine	1 (12.5)	
	Surgery	2 (25)	
	Pediatrics	1 (12.5)	
**Years in working for OHI^a^ websites**			
	1-2 years	4 (50)	
	3-4 years	2 (25)	
	>4 years	2 (25)	

^a^OHI: online health information.

### Feelings Toward the Use of OHI

Participants’ feelings toward the use of OHI were classified into four themes: favoring, disliking, neutral, or having ambivalent feelings toward the use of the OHI.

Participants mentioned various reasons to favor the use of OHI. For instance, a female participant explained that OHI made her feel more comfortable when discussing sensitive topics about her health. She mentioned that she wished to avoid the predictable discomfort of face-to-face interactions with her physicians with potentially embarrassing health complaints. Another 38-year-old male participant added that the ability to hide his physical disability made him feel more self-assured and able to talk with others about his health concern confidently. He noted as follows:

I can use the internet, and no one can see what is behind the screen. The good thing about it is that no one will know what you want to hide about yourself.

A 42-year-old female participant noted as follows:

I prefer the internet websites to discuss sensitive or personal things, and no one will know it is you. You can say what you want without embarrassing yourself in front of your doctor.

In addition, participants mentioned that finding other people with similar health problems gave them a sense of reassurance as well as alleviating their stress and guiding them to make better health decisions.

A 27-year-old female participant noted as follows:

After we found out that my husband had cancer. I was shocked. I felt that my life was ended. I can’t explain how the support I found on the internet health groups helped me to regain my strength again and be able to give him the help and care he needs.

Another group of participants expressed their dislikes regarding the use of OHI. They explained that they felt uncomfortable using the internet, especially in front of younger people owing to their limited computer and internet skills. Many participants who were aged ≥55 years mentioned that they preferred to call their physician when they had any health concerns. When they could not reach a physician, they asked for assistance from a friend or a family member to find the OHI they needed. A 58-year-old female participant noted as follows:

I feel that using the Internet is not for me. I am not very good at technology. That’s why I save my time and ask one of my sons to find it for me...I found this more relaxing.

Other participants expressed some concerns when posting their personal information on private health information websites, which may have commercial agendas. They believe that these websites would use their personal information to promote their products. A 36-year-old female participant noted as follows:

Some health websites may have hidden aims to push you to a particular clinic or specific product they promote. You may find them claim to cure incurable diseases like Hepatitis C or AIDS.

Another group of participants expressed a neutral feeling regarding the use of OHI. *A 47-year-old woman* explained that she sought health information from any available health information resource without preference:

I understand that the health information on the internet is a good thing, but for me, I feel all other health information is similar.

In this theme, many participants expressed positive feelings toward seeking OHI. They mentioned that OHI helped them feel confident and empowered to make decisions regarding their health. However, other participants linked their dislike of OHI to their limited health-related knowledge, limited internet skills, and a lack of trust.

### OHI Seeking Behavior

In this theme, participants explained how their personal experience in accessing and using OHI influenced their behavior toward further use of the internet as a source of health information.

As previously outlined, many participants expressed their preference for using OHI because of its convenience and accessibility. They also mentioned that financial constraints or having a busy schedule encouraged them to prefer OHI in comparison to other, more traditional sources of health information.

A 34-year-old female participant noted as follows:

For me, the most important thing is to avoid transport. I have two baby daughters, so I like to use the internet to find what I want easily.

A 42-year-old male participant noted as follows:

I prefer to find health information from the internet than from any other source of health information. For me, it’s always the first place to go.

Participants mentioned that the limited consultation time in hospitals or clinics encouraged them to seek OHI to find answers to their health enquires. Some participants preferred to seek OHI before their consultation to be able to discuss the information they got with their physicians. Others preferred seeking OHI after their consultation to find supplementary health information or a second opinion regarding their health problems.

A 42-year-old male participant noted as follows:

Sometimes the doctor does not have enough time to discuss or explain everything to me, especially when I see them in the hospital, so I usually look for more information on the Internet after I see him.

Finding trustworthy Arabic health information websites on the internet was a major challenge faced by most of the study participants. Many participants looked for health information on websites run by governmental organizations, as they felt more confident about the reliability of the provided health information. A 47-year-old male participant noted as follows:

We need to understand that not all health information pages are trustable. In my opinion, health websites sponsored by the Government are trustable sources of health information.

In contrast, a 27-year-old male participant mentioned that the accessibility of the internet service and the connection speed represent another constraint to limit an entire subgroup of the population living in rural areas to access web-based health information. He noted as follows:

In my village, the internet is slower and more expensive than it is at the university. If you need to access the internet to find important health advice, you can’t.

In this theme, the study participants mentioned many other factors that encouraged them to seek OHI, such as the convenience of OHI, limited consultation time when seeing a physician, and the variability of health topics on the internet. Another group of participants demonstrated many constraints, which prevented them from using the OHI such as their limited access to internet services and the difficulty in finding trustworthy health information websites.

### Influence of OHI on Participants’ Health

The influence of OHI on participants’ health was categorized into three subthemes; OHI helps users to (1) understand their health concerns, (2) improve their confidence when communicating with their physicians, and (3) achieve higher levels of satisfaction with health services. Many participants mentioned that the use of OHI enhanced their knowledge and understanding of their health concerns more than traditional health information sources, such as leaflets or information provided by physicians. A male participant mentioned that being able to understand his health problem better helped him enjoy a healthier life. Another 27-year-old male participant mentioned that he sought OHI to improve his understanding of his newly diagnosed health condition. He noted as follows:

At least after I read it [refers to the online health information] I know how to ask the doctor about my next steps. If I do not understand my health problem, I will not be able to discuss it with my doctor.

The participants agreed that OHI helped them become more confident when making a health decision, discussing their management options, or sharing their feelings with their physicians or family members. Another participant explained that he kept a list of questions regarding the health information he found on the internet to discuss it with his physician in his next visit. A 29-year-old female participant noted as follows:

After searching the internet for health information, I can say that it has helped me to understand my health problem more and gave me the courage to make better decisions for my treatment.

Most of the participants reported achieving satisfaction with the health information obtained from the internet, especially when they were encouraged by their physicians to seek OHI. They also preferred to use health information from health websites that are recommended by their physicians. A 35-year-old female participant noted as follows:

When I asked my doctor about managing my condition (the participant is diabetic), he encourages me to search the internet. If I find health information, I print it and discuss it with him on my next visit.

A 45-year-old male participant noted as follows:

I feel lucky that my doctor always encourages me to search health information on the internet and I believe this helped me a lot to be more confident about my treatment plan.

In this domain, participants explained how the use of OHI improved their confidence and understanding of their health problems, particularly when they discussed their health management with their physicians. It also has a positive influence on their relationship with their physicians.

### Physicians Interview Findings

Physicians in this study expressed different views based on their experience in providing OHI to Egyptian OHI seekers. We presented physicians’ quotes in Table 3 owing to the small number of participating physicians in this study in comparison to the number of OHI seekers. Their personal experiences and opinions were essential to understand the impact of OHI on the physician-patient relationship. Themes emerging from physicians’ interviews include the accessibility of OHI, good communication, and coordination of care between patients and their physicians, and the active engagement of patients with their management plan has a great impact on the effectiveness of the OHI and a better physician–patient relationship. Many physicians agreed that the benefits of using OHI outweighed its drawbacks when working with Egyptian internet users. They explained that some OHI users simply wanted to be informed about their condition, while others wanted to have full control over all medical decisions. However, this may result in incorrect health decisions or stress to the OHI seekers. A 48-year-old surgeon noted as follows:

To be honest, I love patients’ sense of curiosity about their health and how they want to know more, but not every weight loss is cancer. Their online searches can sometimes go very far resulting in unnecessary stress.

Physicians described the impact of OHI on the physician-patient relationship as a variable. One explained that some physicians advise their patients to trust their expertise and not to use OHI, as they feel that patients are challenging their knowledge when they seek OHI. In contrast, others encourage their patients to seek OHI to become more aware of their health problems and more involved in their health management. Another participant explained that guiding patients toward reliable health information is a part of their responsibilities.

A 48-year-old male participant noted as follows:

Delivery of validated health information through the internet should be our {physicians who provide health information through the internet} priority. We should provide our patients with a trustable source of health information.

A 38-year-old pediatrician noted as follows:

We know that the Internet is accessible, and patients will use it. So, we should probably reduce the harm and refer them to reliable and high-quality health information websites.

In this section, we explain how the physician-patient relationship has changed because of patients’ use of OHI. Therefore, physicians and patients should collaborate in finding reliable health information websites.

**Table 3:** Major themes for physicians’ attitude toward the OHI^a^.

Themes or subtheme and attitude toward OHI			Example quotes
**Accessibility**			
	Positive	“Waiting times in the hospital or clinics in general can be very long. So, when patients search the internet for health advice, they can avoid unnecessary travel time and get the information they need.”	
**Coordination of care**			
	Positive	“Instead of the doctor being the only manager of patient care, internet health has emerged in which patients and their doctors are partners in managing their care.	
	Positive	“the Internet can actually empower patients and enrich the patient–doctor relationship.”	
**Technical competence**			
	Positive	“I believe doctors should know the available sources of OHI and be aware of the updates.”	
	Negative	“We should not assume that all patients know how to use the internet and access reliable health information sources.”	
**Communication**			
	Negative	“Some physicians have concerns regarding the over informed patient.”	
	Negative	“Some patients may give negative impression on their physicians when they discuss the obtained OHI with them. They feel challenged by their patients.”	
	Negative	“I believe the intention to provide online health information was to grant doctors better conditions, to be faster with patients. but with over-informed patients, we spent more time with them to convince them with the treatment options and answer their endless questions.”	
**Engagement**			
	Positive	“patients who read more about their health problem, I found them more engaged in their management plan.”	
	Positive	“I think doctors has an important role which is encouraging patients to read more about their health problem and to take part in their health care plan.”	

^a^OHI: online health information.

## Discussion

### Principal Findings

In this study, we used a qualitative approach to provide an insight into the attitudes of Egyptian internet users and their OHI-seeking behaviors and the perspectives of physicians engaged with OHI. We used a graphic representation to show the key elements of our findings (Figure 1). Overall, the participants expressed a preference for using the internet as a source of health information in comparison with other health information sources, which was consistent with previous studies with both English-and Arabic-speaking internet users [3,12]. Participants provided convenience, anonymity, and accessibility of OHI as reasons for using OHI. Convenience and accessibility were also mentioned as important to young people who search the internet for mental health resources [18]. Notably, we found that traditional sources of health information (eg, physicians and television) are still the primary sources for other health information seekers, especially older adults who prefer personal interaction rather than using the internet, which is consistent with previous studies [2].

In this study, participants explained that OHI helped them understand their health problems more clearly, especially when they had a newly diagnosed health condition. The findings also suggest that OHI provides users with social support that encourages them to further use of health information on the internet. They explained that finding social support from persons with similar health problems facilitated their emotional adjustment and practical abilities to care for and access services for their health or other family members. In similar studies, health advice provided on the internet gave positive feedback on coping with emotional distress especially for OHI seekers with cancer and encouraged them to join the internet health communities [19,20].

Finding social support from patients with similar health problems has been reported in this study to reduce anxiety, especially for patients who are immobile and homebound because of their debilitating illness [21]. In contrast, Fergus et al [22] found that repeated searches for health information on the internet could exacerbate health anxiety for some users, especially in the absence of physicians’ guidance. As reported in the physicians’ interviews, the use of OHI may lead to confusion and cause unrealistic expectations from the obtained OHI, consequently increasing medical litigation against health care providers. Another study found that seeking OHI may increase the cost of health care by adopting inappropriate health advice, making the patients overconcerned about their health, and it could disturb the time efficiency of the physician consultation [23,24].

In this study, participants mentioned that limited internet service and connection speed in their residence were key barriers to prevent them from seeking OHI. Similar to our findings, a study by McCloud et al [25] found that poor internet connections and access restrictions are significant barriers to seek OHI by adults, especially in rural areas. In this study, participants mentioned difficulties in finding trustable Arabic health websites owing to a shortage of this type of information, as they believe that English language is the dominant internet-based language. They explained that there are few certified Arabic medical websites, except websites of international organizations that provide translated content into different languages. Another study explained that the availability of OHI in the native language may enhance the utility of the internet for health information [26]. In addition, the quality of Arabic language health information websites was questioned by Alhajj et al [27], who concluded that the quality of web-based Arabic health information was poor regardless of being readable by internet users. The development of more credible Arabic websites offering more evidence-based and regularly updated information is recommended [28].

In this study, physicians expressed a general positive attitude toward the effects of OHI on users’ health outcomes. They explained that sharing and discussing the obtained OHI with their patients will help internet users be more confident about their health decisions. Laugesen et al [29] explained that physicians have a greater impact on patient’s health outcomes and compliance than internet health information. Concerns expressed by physicians in this study about the quality of health information on the internet and access to reliable websites followed similar studies [12,30]. Broom et al [24] recommended that to promote the impact of OHI on health, physicians should consider it as a complementary tool, rather than reflecting a breakdown in their authority or status. Hence, Egyptian health care providers need to take actions to ensure the dissemination of correct and reliable health information on the internet that matches the needs of Egyptian OHI seekers.

### Limitations

Participants of this study were Egyptian adults who had already used the internet as a source of health information. We did not explore the perspectives of Egyptians who did not use the internet for health information. In addition, the personal characteristics of the participants were that we did not medically verify their general health status as it was self-assessed. Although participant numbers are usually small for this type of qualitative research, a larger number of participants could have uncovered a wider range of perspectives on the topic of study. Therefore, further research with a larger, purposive sample of Egyptian internet users would be valuable. In addition, we could not medically verify participants’ general health status as it was self-assessed, and we did not have access to their medical records.

### Conclusions

Our study is the first qualitative study to evaluate the attitude of Egyptians toward using OHI and how it influences their health behavior and their relationship with their physicians. The study participants demonstrated numerous benefits and challenges of using Arabic health information websites. Participants explained how OHI improved their knowledge and confidence, particularly when making health decisions. In addition, the findings from this study point to a range of concerns regarding the quality of the provided OHI and the limited availability of reliable Arabic health information websites. We recommend that health care providers provide more guidance and support to Egyptian OHI seekers to find good-quality health information websites. The responsible authorities should also ensure the availability of reliable Arabic language health websites.

## Abbreviations

FGD: focus group discussion
OHI: online health information
PHI: personal health information
